# Consistent Killers: Conservation of Thrombin-Like Action on Fibrinogen by Bushmaster (*Lachesis* Species) Venoms Underpins Broad Antivenom Cross-Reactivities

**DOI:** 10.3390/toxins17050224

**Published:** 2025-05-02

**Authors:** Lee Jones, Bryan G. Fry

**Affiliations:** Adaptive Biotoxicology Lab, School of the Environment, University of Queensland, St. Lucia, QLD 4072, Australia

**Keywords:** *Lachesis*, Venom, Coagulotoxicity, Antivenom

## Abstract

Snakebite represents a significant public health challenge in Central and South America, with *Lachesis* (Bushmaster) species posing unique clinical challenges due to their severe envenomation effects arising from a combination of potent venom and copious venom yields. Using in vitro coagulation assays, we analyzed the coagulotoxic venom effects from four distinct localities: *L. muta* from Surinam and French Guiana and *L. stenophrys* from Costa Rica and Panama. This study examined the venom’s impact on human plasma and fibrinogen and evaluated the efficacy of two regionally available antivenoms (PoliVal-ICP and Antivipmyn-Tri) in neutralizing the pathophysiological effects. Our results demonstrated a remarkable consistency in the pseudo-procoagulant venom activity (also known as: thrombin-like) across different species and localities. Antivenom efficacy testing revealed that both the PoliVal-ICP and Antivipmyn-Tri antivenoms effectively neutralized the venom effects across localities for both species, with the ICP antivenom showing the highest neutralization capacity. These toxicology findings highlight the biochemical conservation of venom composition across *Lachesis* species which underpins effective cross-neutralization in antivenom treatment.

## 1. Introduction

Snakebites represent a significant yet neglected public health challenge throughout Central and South America, with approximately 60,000 envenomations occurring annually across the region [1]. Rural and indigenous communities bear a disproportionate burden of snakebite morbidity and mortality due to their proximity to snake habitats, occupational exposure during agricultural activities, and limited access to timely healthcare and antivenom therapy. The species primarily responsible for the majority of medically significant snakebites in these regions belong to the family Viperidae, particularly of the genera *Bothrops* (lancehead pit vipers), *Crotalus* (rattlesnakes), and *Lachesis* (bushmasters) [2,3].

While *Bothrops* reportedly account for 90% of bites in Latin America [4], envenomations by *Lachesis* species are particularly concerning due to their severe clinical outcomes [5,6,7,8,9]. Following envenomation, victims experience local pain, edema, hemorrhage, and myonecrosis, symptoms similar to those observed in bothropic envenomings [10]. The distinct feature of bites by *Lachesis* taxa is the prominence of autonomic disturbances, including hypotension, bradycardia, abdominal pain, diarrhea, and profuse sweating, symptoms rarely seen with such severity in other pit viper envenomations [6,7,8,11]. Coagulopathy develops quickly and may manifest as gingival bleeding, hematuria, and persistent bleeding from the fang punctures or venipuncture sites, reflecting the potent fibrinogen-depleting activity of *Lachesis* venoms [9]. *Lachesis* poses unique clinical challenges across its extensive range, as these extremely large-bodied pit vipers can deliver substantial venom volumes, and their habitat in remote forested regions frequently coincides with areas of limited healthcare access [12].

Despite *Lachesis* species inhabiting a wide geographic range, from Central America extending though South America, their venoms have shown remarkably similar compositional and pharmacological profiles between and within species [13,14]. The coagulotoxic effects that characterize *Lachesis* envenomation stem from a sophisticated arsenal of hemotoxic components that have been studied through functional and proteomic analyses [13,15,16,17,18]. Research has identified multiple enzymes in *Lachesis* venoms, including well-characterized kallikrein-like serine protease toxins (SVSPs) such as TLE-B and TLE-P in *L. muta* [19] and stenoxobin in *L. stenophrys* [20], which are isolated ‘thrombin-like’ enzymes that aberrantly cleave fibrinogen to induce the formation of unstable fibrin clots in a pseudo-procoagulant manner. These fibrin clots have short half-lives, with their rapid breakdown leading to a depletion of fibrinogen levels, thereby contributing to a net anticoagulant effect. Beyond direct effects on fibrinogen, these venoms contain diverse components that interfere with platelet function, including C-type lectin-like proteins that either induce or inhibit platelet aggregation [21,22,23]. The hemorrhagic activity observed in envenomations is further enhanced by snake venom metalloproteinases (SVMPs) that degrade basement membrane components, compromising vascular integrity [24,25]. This complex interplay between fibrinogen-depleting enzymes, factors that inhibit clotting enzymes, and hemorrhagins results in the severe coagulopathy characteristic of *Lachesis* envenomation, producing a multifaceted disruption of hemostasis that remains challenging to treat despite advances in antivenom therapy [13].

Due to the rapid onset of local and systemic clinical manifestations, treatment relies principally on the timely administration of appropriate antivenoms [26,27,28]. Given the medical significance of *Lachesis* envenomations, they are often represented in the immunizing mixture of antivenoms. In Central America, the polyvalent antivenom from Instituto Clodomiro Picado (ICP) includes *L. stenophrys* immunogens and shows good clinical efficacy and cross-reactivity against all *Lachesis* species tested [3,17,29]. South American antivenoms including *L. muta*/*L. muta rhombeata* are produced by several institutions including Instituto Butantan (São Paulo, Brazil), Instituto Nacional de Higiene (Caracas, Venezuela), Instituto Bioclon (Mexico City, Mexico), and Instituto Nacional de Salud (Bogotá, Colombia), with variable cross-neutralization profiles reflecting the underlying venom variation [3].

Antivenom therapy remains the cornerstone of managing *Lachesis* envenomation, and continued testing across the extensive range of *Lachesis* remains vital to further develop our understanding of geographical ranges in efficacy of antivenom and cross-neutralization between species. This research aims to bridge the knowledge gap by furthering our understanding between localities of *Lachesis* venom’s ability to act directly on fibrinogen, as well as the neutralization scope of the Mexican Antivipmyn-Tri antivenom and the ICP antivenom from Costa Rica.

## 2. Results and Discussion

**Figure 1:**
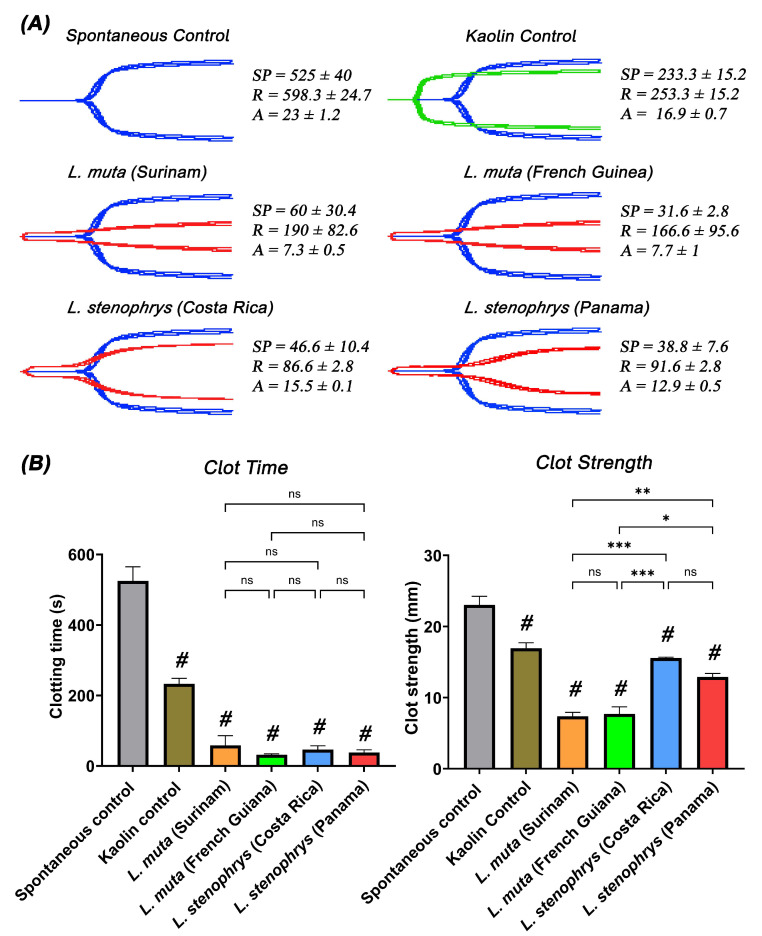
*Lachesis* venom effects on human plasma in comparison to the spontaneous control. (**A**) Fibrinogen TEG traces showing blue traces represent the spontaneous controls; green traces represent the kaolin control, and red traces represent samples incubated with venoms. SP = split point, time taken until a clot is formed (min). R = time taken until clot reaches 2 mm + (min). A = amplitude, indicating clot strength (mm). (**B**) Bar graphs showing clot time (SP) and clot strength (A) with one-way ANOVA with Dunnett’s multiple comparisons tests for significance compared to the spontaneous control, whereby # indicates that the sample was significantly different to the spontaneous control (*p* < 0.01), and * (*p* < 0.1) ** (*p* < 0.01), and *** (*p* < 0.001 show significance between samples. While “ns” is non-significant (*p* > 0.1). Assays were performed in triplicate (*n* = 3) with data representing the mean ± SD.

This study analyzed in vitro coagulotoxic venom activity in two localities of *Lachesis muta* (French Guiana, Surinam) and *Lachesis stenophrys* (Costa Rica, Panama). Clotting assays showed that the venoms possessed the ability to quickly clot human plasma (Figure 1). The venom from both localities of *Lachesis muta* and *L. stenophrys* were significantly faster than the kaolin control, indicating potent pseudo-procoagulant effects. In addition, we show no significant differences between and within samples for both plasma and fibrinogen clotting. Thromboelastography shows that these clots are significantly weaker than the spontaneous control, which is indicative of the pseudo-procoagulant (i.e., thrombin-like) activity characteristic of *Lachesis* venoms. On human plasma, the *Lachesis stenophrys* venoms from Costa Rica and Panama both induced a weak clot before increasing in strength, as demonstrated by the R value. This activity was not seen in the venom of the Surinam and French Guinea samples of *L. muta*, in which clots remained weak throughout the 30 min experiment. This is suggestive of incomplete action on fibrinogen, with spontaneously generated thrombin acting on the remainder.

**Figure 2:**
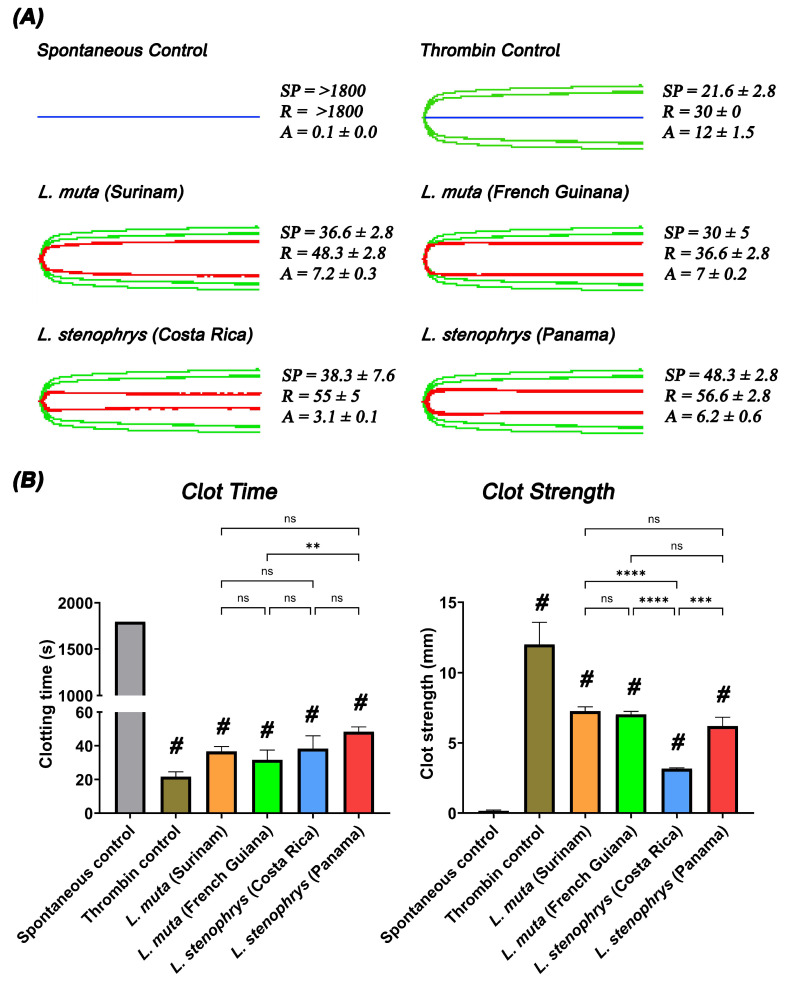
*Lachesis* venom effects on human fibrinogen in comparison to the thrombin control. (**A**) Fibrinogen TEG traces showing blue traces represent the spontaneous controls; green traces represent the thrombin control, and red traces represent samples incubated with venoms. SP = split point, time taken until a clot is formed (min). R = time taken until clot reaches 2 mm + (min). A = amplitude, indicating clot strength (mm). (**B**) Bar graphs showing clot time (SP) and clot strength (**A**) with one-way ANOVA with Dunnett’s multiple comparisons tests for significance compared to the spontaneous control, whereby # indicates significance to the spontaneous control (*p* < 0.01); ** (*p* < 0.01), *** (*p* < 0.001), and **** (*p* < 0.0001) show significance between samples. While “ns” is non-significant (*p* > 0.1). Assays were performed in triplicate (*n* = 3) with data representing the mean ± SD.

Further TEG studies on isolated human fibrinogen confirmed the potent pseudo-procoagulant (thrombin-like) effects, with venoms continuing to display weak clots in fibrinogen compared to the thrombin control (Figure 2). We show no inter- or intraspecific differences with regard to clotting times, with each venom inducing clots similar to the thrombin control. Interestingly, the venoms from both species of *Lachesis* showed the opposite effect than what was observed in plasma, where *L. stenophrys* showed significantly weaker clots in comparison to both *L. muta* localities. Previous studies have shown that isolated thrombin-like enzymes from *Lachesis* venoms specifically cleave the Aα chain of fibrinogen with minimal activity toward the Bβ chain while leaving the γ chain intact [19,25]. The weak, aberrant clots are easily broken down, contributing to the coagulopathy often observed in *Lachesis* envenomation.

**Figure 3:**
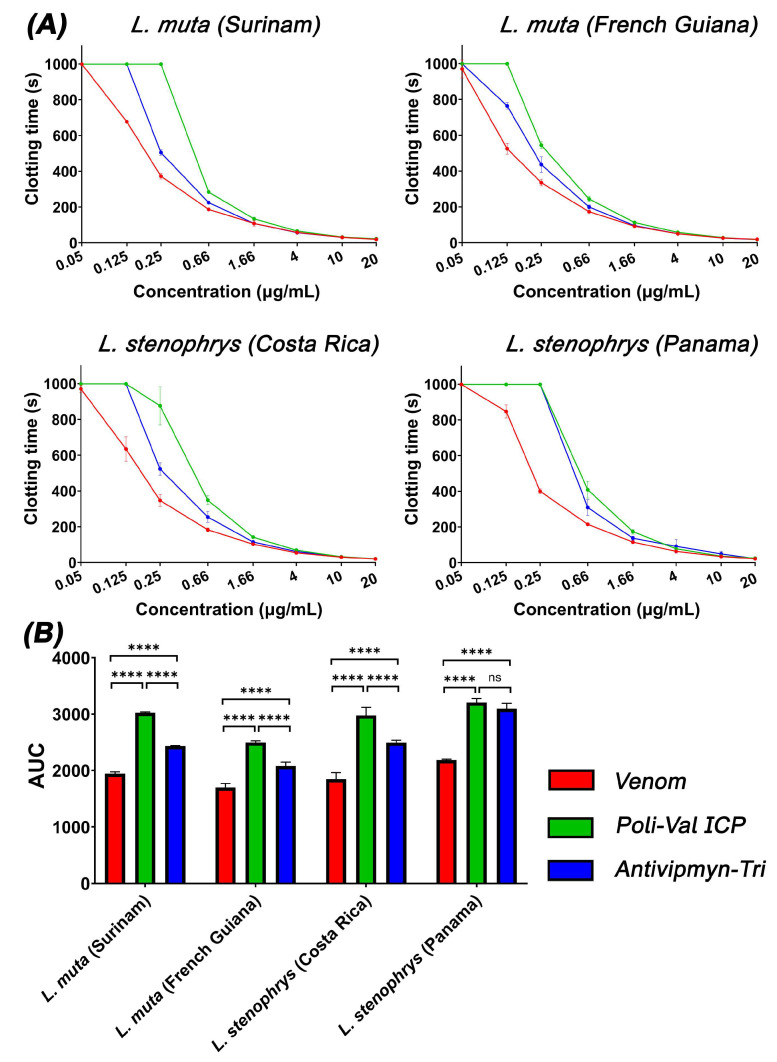
Neutralization of fibrinogen clotting venom effects via the PoliVal-ICP antivenom. (**A**) The 8-point concentration–response curves showing the fibrinogen clotting time of venom only (red), venom + ICP antivenom (green), and venom + Antivipmyn-Tri antivenom (green). Assays were performed in triplicate (*n* = 3) with data representing the mean ± SD. (**B**) Area under the curve bar graphs showing venom (red) and venom + the ICP antivenom (green) and venom + the antivipmyn-tri antivenom (blue). Values were analyzed using one-way ANOVA with multiple comparisons to compare between samples, with **** indicating *p* < 0.0001. While “ns” is non-significant (*p* > 0.1).

Eight-point dilution curves were used to determine the efficacy of the antivenoms against the fibrinogen clotting activity from two localities of *Lachesis muta* (Surinam and French Guinea) and *Lachesis stenophrys* (Costa Rica and Panama) (Figure 3A). AUC values show that both the ICP and Antivipmyn-Tri antivenoms were overall effective in neutralizing the fibrinogen clotting activity across all samples (Figure 3B). The antivenom effects were dose-dependent, as demonstrated by the poorly neutralized venom at concentrations above 1.6 μg/mL. The ICP antivenom showed higher neutralization in comparison to Antivipmyn-Tri, with the exception of the Panama sample of *L. stenophrys*, in which the antivenoms showed statistically equipotent neutralization of venom effects.

Paraspecificity is well documented in the ICP antivenom, yielding positive results across many localities and species not included in the immunizing mixture [29,30,31,32,33]. In contrast, research on Antivipmyn-Tri remains scarce, with existing studies bias towards *Bothrops* and *Crotalus* [34,35,36,37]. In French Guiana, while Antivipmyn-Tri is used to treat crotalid envenomation, however, the clinical efficacy remains unclear. Heckmann [38] reports no benefit in recovery when administrating the Antivipmyn-Tri antivenom following crotalid bites, whereas Resiere [39] documents the effective reversal of crotaline envenomation-induced coagulation disorders. These divergent clinical observations align with in vitro coagulation assays, which revealed low neutralizing potential across multiple localities of *Bothrops atrox* [33]. Although the ICP antivenom demonstrated higher neutralization for the French Guiana locality in our study, Antivipmyn-Tri still exhibited significant venom effect neutralization of venom effects, at least for *Lachesis* venoms. Resiere’s [39] research corroborated these findings by comparing the efficacy of the ICP and Antivipmyn-Tri antivenoms against French Guianan *B. atrox* and confirming that while both antivenoms were effective, the ICP antivenom showed higher neutralization capabilities.

Factors contributing to differential antivenom neutralization outcomes include the immunizing mixtures used to make the antivenoms, as well as the molecular characteristics of the antivenoms. The PoliVal-ICP antivenom (Instituto Clodomiro Picado, Costa Rica) contains an immunizing mixture with equal parts *Bothrops asper*, *Crotalus simus*, and *Lachesis stenophrys*. In contrast, the Antivipmyn-Tri antivenom (Instituto Bioclon; Mexico) contains *Bothrops asper*, *Crotalus durissus*, and *Lachesis muta*. In addition to the immunizing mixture, these antivenoms also differ physiochemically. The ICP antivenom utilizes whole immunoglobulin (IgG) molecules, while Antivipmyn-Tri comprise refined F(ab)^2^ fragments. Further research should be conducted in order to determine the cross-neutralization efficacy of both antivenoms across different localities and sympatric species.

Despite the different *Lachesis* species used with ICP and Antivipmyn-Tri (*L. stenophrys* and *L. muta*, respectively), both antivenoms were overall effective in neutralizing the venom effects from each species. Effective cross-neutralization across geographic localities within *Lachesis* species may be attributed to the similarity of venom components [16,17,18]. Previous research highlighting the compositional and pharmacological conservation across *Lachesis* venoms, resulting in the addition of a single representative for the genus, provides adequate paraspecific protection against conspecific venom activity [3]. Our results support this for the venoms’ actions on fibrinogen, which is one of the major clinically relevant coagulopathic actions of these venoms.

*Lachesis* shows significant variation in its geographic distributions, with some species occupying extensive ranges and others confined to restricted areas. Despite this broad range and the isolation of some populations, their venoms exhibit remarkable similarity in their functional coagulotoxic effects. Our results corroborate this, with each species and locality tested showing the same pseudo-procoagulant venom activity to those previously reported [3,20,40,41]. Pseudo-procoagulant (thrombin-like) activity is a basal trait of crotaline venoms, as characterized in *Lachesis*, *Bothrops*, *Crotalus*, *Agkistrodon*, *Atropoides*, *Cerrophidion*, *Metlapilcoaltus*, and *Porthidium* [31,42,43]. The selective pressures driving the amplification in these coagulotoxic enzymes in the venoms likely relate to prey specificity and the effectiveness of inducing consumption coagulopathy. Evidence from natural history and feeding ecology studies suggest that *Lachesis* species, as generalist predators of rodents and other small vertebrates [44], have evolved venom optimized for rapid prey immobilization. The evolution of thrombin-like enzymes in this genus likely reflects the effectiveness of subjugating mammalian prey, resulting in low coagulotoxic variation between species. While we did not test for specificity towards mammalian prey, this provides an avenue for future research to further understand the ecology and evolution of these snakes.

While adult *Lachesis* species do not show high levels of inter- or intraspecific compositional or functional venom variation, there has been ontogenetic shifts reported for *L. stenophrys* [18], such as transitioning the venom profile from serine protease-rich profiles in newborns and juveniles to predominantly SVMP-rich venom in adults. However, it was not determined whether this compositional change is reflected by functional differences across life stages. Further research should investigate the potential ontogenetic changes in venom composition and functional activity in other *Lachesis* species as such age-dependent variations may affect antivenom efficacy. Further understanding of the venom development and evolution of *Lachesis* may also inform antivenom production by determining the cross-reactivity of the adult venom phenotype to represent all life stages.

In conclusion, this research emphasizes the functional similarity across two different *Lachesis* species and geographic localities. We show the effective neutralization of thrombin-like venom effects by both antivenoms, particularly the Polival-ICP antivenom. Future research should focus on investigating ontogenetic venom changes across more *Lachesis* species, exploring the ecological and evolutionary drivers of venom composition, and further evaluating antivenom efficacy across broader geographic ranges. An important caveat is that the current work investigated the effect of antivenom using preincubation conditions. While this method is effective in ascertaining cases of antivenom failure, since if it does not effectively neutralize venom under such idealized circumstances, there is little chance of it working in the dynamic system of the living organism. However, in vivo follow-up studies are necessary to confirm the in vitro positive results revealed in the current study. Understanding these nuanced venom characteristics is critical for improving snakebite treatment strategies in regions containing *Lachesis* species.

## 3. Methods

### 3.1. Venom, Antivenom, and Plasma

All human plasma work was conducted under University of Queensland Biosafety Committee Approval # IBC/149B/SBS/2016 (20 September 2023), UQ Human Ethics Approval #2016000256 (9 May 2024), and Australian Red Cross Research Agreement #16- 04QLD-10 (2 February 2025). Venoms were purchased from licensed biological supply company Latoxan, France. Four venoms were used in this study: *Lachesis muta* (Surinam), *Lachesis muta* (French Guiana), *Lachesis stenophrys* (Costa Rica), and *Lachesis stenophrys* (Panama). All plasma was stored at −80°C until thawed for use. This study used two antivenoms: the PoliVal-ICP antivenom (Instituto Clodomiro Picado; San José, Costa Rica), which contains an immunizing mixture with equal parts *Bothrops asper*, *Crotalus simus*, and *Lachesis stenophrys*, and the Antivipmyn-Tri antivenom (Instituto Bioclon; Mexico City, Mexico), which contains *Bothrops asper*, *Crotalus durissus*, and *Lachesis muta*.

### 3.2. Coagulotoxicity Assays

In order to understand the coagulotoxic effects of the venom, a Stago STA-R Max hemostasis analyzer robot was used to perform coagulation assays. Human plasma was thawed in a 37 °C water bath before being placed into the machine for testing. The 1 mg/mL venom stocks were further diluted to a 1:10 working stock using Owren–Keller (OK) buffer. For concentration curves, the machine automatically performed a series of dilutions to the 0.1 mg/mL working stock (μg/mL: 0.05, 0.125, 0.25, 0.66, 1.66, 4, 10, and 20). For the 1:1 dilution, 50 μL of the working stock was added to 50 μL of phospholipid, 50 μL of calcium, and 25 μL of OK buffer and then incubated for 120 s. Finally, the machine then added 75 μL of plasma, which then automatically measured the time until clot formation. For antivenom treatments, a 50 mg/mL antivenom concentration replaced the OK buffer. If a clot had not formed within the maximum recording time (999 s), the machine automatically stopped. The spontaneous clotting time of human plasma was used as a negative control and as a determinate of plasma health. All venoms were tested against the PoliVal-ICP and Antivipmyn-Tri antivenoms in triplicate.

### 3.3. Thromboelastography (TEG)

To further understand clotting within human plasma and fibrinogen, TEG was used to determine the timing and strength of clots formed through the addition of *Lachesis* venoms. Experiments were performed using methods previously validated in the Venom Evolution Lab. A total of 7 µL of 1 mg/mL venom was added to 72 µL of CaCl2, 72 µL of phospholipid, and 20 µL of OK buffer, followed by 189 µL of plasma/fibrinogen, and then run immediately for 30 min to allow for clot formation. Raw data can be viewed in the Supplementary Materials (File S1).

### 3.4. Statistical Analysis

All assays were performed in triplicate (*n* = 3). Data were analyzed using the GraphPad prism 10.4.1 software. For the comparison of antivenom efficacy in neutralizing venom activity, the area under the curve (AUC) was calculated for venom curves and venom + antivenom curves, as well as the percentage increase in AUC.

## Data Availability

The original contributions presented in this study are included in the article/Supplementary Material. Further inquiries can be directed to the corresponding author(s).

## Supplementary Materials

The following supporting information can be downloaded at: https://www.mdpi.com/article/10.3390/toxins17050224/s1, Supplementary File S1, raw data.
